# Adiponectin: A Promising Target for the Treatment of Diabetes and Its Complications

**DOI:** 10.3390/life13112213

**Published:** 2023-11-16

**Authors:** Mahmuda Begum, Mayank Choubey, Munichandra Babu Tirumalasetty, Shahida Arbee, Mohammad Mohabbulla Mohib, Md Wahiduzzaman, Mohammed A. Mamun, Mohammad Borhan Uddin, Mohammad Sarif Mohiuddin

**Affiliations:** 1 Department of Internal Medicine, HCA-St David’s Medical Center, 919 E 32nd St, Austin, TX 78705, USA; mahmuda_b@yahoo.com; 2 Department of Foundations of Medicine, NYU Grossman Long Island School of Medicine, 101 Mineola Blvd, Mineola, NY 11501, USA; choubeymayank48@gmail.com (M.C.); tmunichandrababu@gmail.com (M.B.T.); wahid.bmb.cu@gmail.com (M.W.); 3 Institute for Molecular Medicine, Aichi Medical University, 1-Yazako, Karimata, Aichi, Nagakute 480-1103, Japan; shahida.arbee.chobi@gmail.com; 4 Julius Bernstein Institute of Physiology, Medical School, Martin Luther University of Halle-Wittenberg, Magdeburger Straße 6, 06112 Halle, Germany; mohib_nsu007@yahoo.com; 5 CHINTA Research Bangladesh, Savar 1342, Bangladesh; mamunphi46@gmail.com; 6 Department of Public Health and Informatics, Jahangirnagar University, Savar 1342, Bangladesh; 7 Department of Pharmaceutical Sciences, North South University, Dhaka 1229, Bangladesh; mohammad.uddin@northsouth.edu

**Keywords:** adiponectin, AdipoRs, diabetes, pancreatic islets, single-cell RNAseq

## Abstract

Diabetes mellitus, a chronic metabolic disorder characterized by hyperglycemia, presents a formidable global health challenge with its associated complications. Adiponectin, an adipocyte-derived hormone, has emerged as a significant player in glucose metabolism and insulin sensitivity. Beyond its metabolic effects, adiponectin exerts anti-inflammatory, anti-oxidative, and vasoprotective properties, making it an appealing therapeutic target for mitigating diabetic complications. The molecular mechanisms by which adiponectin impacts critical pathways implicated in diabetic nephropathy, retinopathy, neuropathy, and cardiovascular problems are thoroughly examined in this study. In addition, we explore possible treatment options for increasing adiponectin levels or improving its downstream signaling. The multifaceted protective roles of adiponectin in diabetic complications suggest its potential as a novel therapeutic avenue. However, further translational studies and clinical trials are warranted to fully harness the therapeutic potential of adiponectin in the management of diabetic complications. This review highlights adiponectin as a promising target for the treatment of diverse diabetic complications and encourages continued research in this pivotal area of diabetes therapeutics.

## 1. Introduction

Diabetes mellitus (DM) is one of the most ancient illnesses known to humans. Around 3000 years ago, it was first mentioned in an Egyptian text. In recent decades, there has been a significant surge in the global prevalence of diabetes and its associated metabolic complications. The difference between type 1 and type 2 diabetes was established in 1936 [1]. The frequency of type 2 diabetes mellitus (DM), a chronic metabolic condition, has been continuously rising worldwide. According to the World Health Organization, the global diabetic population is steadily rising, currently estimated at 422 million individuals [2]. T2DM is characterized by dysregulation of carbohydrate, lipid, and protein metabolism; which occurs from decreased insulin secretion, insulin resistance, or a combination of the two; usually occurs later in life; and is frequently linked to lifestyle factors [3]. Among the three main groups of diabetes, which include type 1 diabetes mellitus (T1DM) and gestational diabetes, type 2 diabetes is the most prevalent form [4]. The primary cause of T2DM is a gradual decline in insulin secretion by pancreatic β-cells, typically occurring against a backdrop of pre-existing insulin resistance in skeletal muscle, liver, and adipose tissue [5,6]. According to the World Health Organization (WHO), diabetes stands as a primary contributor to conditions such as blindness, kidney failure, heart attacks, stroke, and lower limb amputations [7]. Diabetes is linked to both microvascular (diabetic retinopathy, neuropathy, and nephropathy) and macrovascular (cardiovascular disease or CVD) complications [8]. Individuals with diabetes face an elevated risk of CVD, encompassing conditions like coronary heart disease (CHD) [9], hypertension, increased levels of low-density lipoprotein-cholesterol (LDL), and obesity [5,10].

Adiponectin, the most prevalent peptide released by adipocytes, plays a prominent role in the intricate connection between adiposity, insulin resistance, and inflammation [11]. Levels of adiponectin are inversely correlated with adiposity, meaning an increase in body fat reduces adiponectin levels and a reduction in fat accumulation increases adiponectin levels [12]. Adiponectin shows its biological action through several mechanisms such as enhancing insulin sensitivity in the peripheral cells [13,14,15], anti-inflammatory actions by reducing the production of inflammatory molecules [16], breakdown of fatty acids and inhibiting the production of fatty acids in the liver [17], maintaining the health and flexibility of blood vessels [18,19], etc. Adiponectin also plays a role in appetite regulation and energy expenditure [20,21].

Today, there are several treatment options for T2DM. However, treating T2DM is very challenging due to its complex nature and the diversity of factors involved. T2DM is a progressive disease as the production of insulin is reduced day by day irrespective of the treatment of diabetes. Insulin resistance is another challenge in treating diabetes. Lifestyle modifications including diet and exercise play a crucial role in diabetes. However, sustaining these alterations can be arduous. Moreover, chronic conditions like T2DM have significant effects on emotional health. 

Recent studies have shown that a reduction in plasma adiponectin concentration is closely associated with the development of type 2 diabetes mellitus (T2DM) and obesity [22,23]. Both animal and experimental research have demonstrated that adiponectin enhances insulin sensitivity, suggesting that it may serve as a preventive measure against the onset of T2DM [24].

In the current review, we will discuss recent advancements in studying the pathophysiological functions of adiponectin and its receptors in relation to insulin resistance, type 2 diabetes, and metabolic syndrome.

## 2. Adiponectin: An Overview

**Figure 1:**
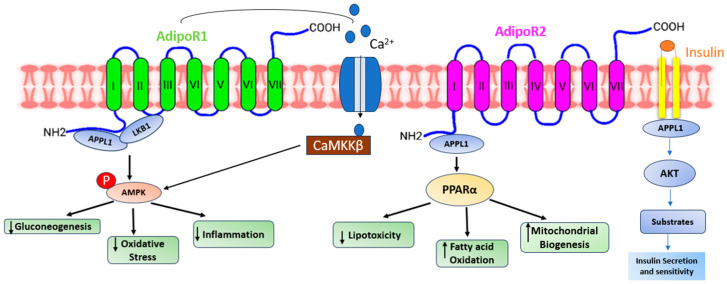
A presentation demonstrating the diverse pathways through which adiponectin receptors exert their functions. Adiponectin engages with its receptors to initiate various signaling pathways. AdipoR1 enhances calcium influx, leading to the activation of Ca^2+^/calmodulin-dependent protein kinase kinase β (CaMKKβ) and subsequent downstream kinases. AdipoR1- and R2-dependent signaling are mediated by adaptor protein phosphotyrosine interaction (APPL) 1, which allows LKB1 to translocate from the nucleus to the cytoplasm and activate AMPK, ceramidase activity, and peroxisome proliferator-activated receptor-alpha (PPAR-α). Activation of AMPK reduces gluconeogenesis, oxidative stress, and inflammation. On the other hand, activation of PPAR-α reduces lipotoxicity and inflammation and increases fatty acid oxidation. All these effects ultimately improve glycemic status.

Adiponectin, alternatively referred to as AdipoQ, APM1, or ACRP30, is a single-chain adipokine composed of 244 amino acids, possessing a molecular weight of around 26 kilodaltons (kDa) secreted by white adipose tissue. The adiponectin protein is encoded by the AdipoQ gene located on the chromosome locus 3q27. Adiponectin consists of several distinct structural components. It includes an NH2-terminal hyper-variable region, a collagenous domain consisting of 22 Gly-XY repeats, and a COOH-terminal C1q-like globular domain. When secreted into the bloodstream, adiponectin forms three oligomeric complexes: a trimer, a hexamer, and a high molecular weight multimer [25]. Adiponectin primarily binds to seven transmembrane receptors known as AdipoR1 and AdipoR2 to regulate a range of physiological functions including whole-body energy balance, inflammatory responses, insulin sensitivity, and the process of fat metabolism [26]. Unlike traditional G-protein coupled receptors, these receptors possess a cytoplasmic NH2 terminus and an extracellular COOH terminal domain. AdipoR1 is most abundantly expressed in skeletal muscle, whereas AdipoR2 is predominantly expressed in the liver [27]. In humans and mice, AdipoR1 is situated on chromosome 1p36.13-q41, while AdipoR2 is found on chromosome 12p13.31 and 6 F1. The molecular structure of both forms of the receptor exhibits significant homology, featuring an internal N-terminus and an external C-terminus [28]. AdipoR1 and AdipoR2 (Figure 1) are adiponectin receptors that stimulate AMP-activated kinase (AMPK) and PPAR activity, regulating glucose and lipid metabolism. Adiponectin-induced complete AMPK activation requires both Ca^2+^/CaMKK and AMP/LKB1 [29].

**Figure 2:**
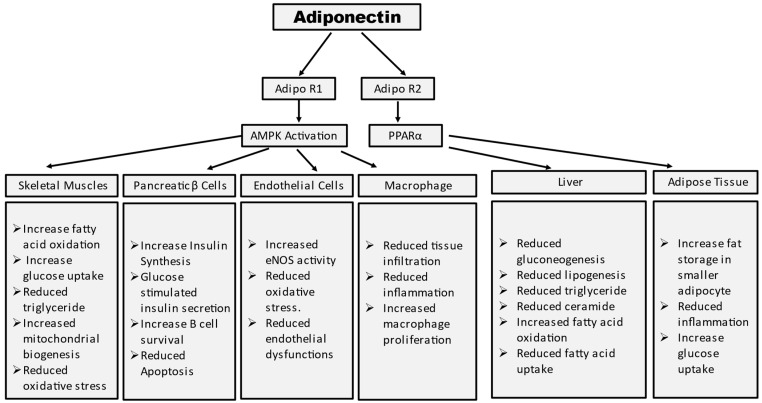
Summary of tissue-specific functions of adiponectin. Mechanism of adiponectin actions in prevention of insulin resistance and diabetes.

Adiponectin stimulates glucose uptake and fatty acid oxidation in skeletal muscle after binding to AdipoR1, which is mediated by the recruitment of the adaptor protein with the pleckstrin homology domain, phosphotyrosine domain, and leucine zipper domain (APPL). APPL binding to the intracellular domain of AdipoR1 activates Rab5, a small GTPase that enhances GLUT4 membrane translocation and glucose absorption in muscle. APPL also binds to PI3 kinase and Akt, showing that adiponectin can boost insulin signaling as well [30]. The interaction between APPL and AdipoR1 activates AMP-activated protein kinase (AMPK), which inhibits acetyl-CoA carboxylase (ACC) and promotes fatty acid oxidation—adipoR-mediated activation of AMPK leads to higher fatty acid oxidation and decreased obesity. AMPK activation increases glucose absorption and lactate generation in muscle while suppressing gluconeogenesis. Together, the adiponectin signaling pathways underscore the relevance of adiponectin in glucose and lipid metabolism (Figure 2) [31].

Adiponectin activates and enhances the production of PPAR ligands via AdipoR2, as well as fatty acid combustion and energy consumption. This is accomplished in part by enhanced expression of the ACO and UCP genes, which include the peroxisome proliferator response element (PPRE) in their promoter regions [32].

## 3. Adiponectin Pathway Regulation

Adiponectin regulation is a complicated combination of genetic, hormonal, and environmental factors. The adiponectin regulatory mechanism centers mostly around the expression and release of adiponectin from adipocytes.

### 3.1. Genetic Factors

Adiponectin is mostly generated in white adipose tissue by mature adipocytes. Originally assumed to be expressed solely by adipose tissue, it is now widely documented that adiponectin is generated and released by a variety of cell types, including skeletal and cardiac muscles [33,34]. The normal range of adiponectin in human plasma is 2–20 μg/mL. Adiponectin levels are also regulated by genetic variations in the ADIPOQ gene, which encodes adiponectin. Some genetic polymorphisms are linked to increased or decreased adiponectin production [35]. The adiponectin gene, situated on chromosome 3q27, corresponds to a susceptibility locus for diabetes. Various cross-sectional studies have demonstrated an association between single-nucleotide polymorphisms (SNPs) within the adiponectin gene (ADIPOQ) and diabetes. These SNPs within ADIPOQ play a crucial role in evaluating the link between common genetic variants, adiponectin levels, and the risk of diabetes. Notably, two common SNPs, rs2241766 and rs1501299, have shown significant correlations with type 1 diabetes mellitus, contributing to a distinct haplotype block [36,37,38,39,40,41].

### 3.2. Insulin Sensitivity

Insulin sensitivity is one of the most important determinants of adiponectin levels. Higher levels of adiponectin correspond to greater insulin sensitivity. On the other hand, insulin sensitivity is reduced in several diseases such as diabetes and obesity and the adiponectin level also decreases [42]. A team of researchers from the Karolinska Institutet in Stockholm, Sweden conducted a study involving 942 men. They found a robust correlation between insulin sensitivity and three ADIPOQ variants—namely, rs17300539, rs3774261, and rs6444175. In obese individuals, lower serum adiponectin levels were observed compared to those in normal, healthy individuals [43].

### 3.3. Inflammatory State

The levels of adiponectin in the bloodstream decline following an elevation in proinflammatory cytokines like TNF-α and IL-6, along with endothelial reticulum stress and adipocyte hypertrophy. This phenomenon is associated with conditions linked to expanded adipose tissue, including obesity, type 2 diabetes mellitus (T2DM), cardiovascular disease, and metabolic syndrome [44].

### 3.4. Adipose Tissue Distribution

The distribution of adipose tissue affects the levels of adiponectin. Subcutaneous adipose tissue is associated with higher adiponectin levels compared to visceral fat [45].

### 3.5. Diet and Nutritional Factors

Several dietary components such as omega-3 fatty acids and polyphenols are regulated by the production of adiponectin [46]. A group of researchers from Universidade de São Paulo, São Paulo, Brazil observed in a double-blind, placebo-controlled, 2-month clinical trial with 80 individuals that supplementation of ω-3 fatty acid showed an increase in serum adiponectin [47].

### 3.6. Physical Exercise

Routine physical activities, particularly aerobic exercises like jogging or cycling, along with resistance training, can elevate adiponectin levels. Exercise also improves insulin sensitivity, which is associated with adiponectin secretion [48].

### 3.7. Hormonal Regulation

Leptin, another hormone secreted by adipose tissue, can affect adiponectin levels by opposing effects on metabolic regulation [49]. Insulin can stimulate adiponectin production and secretion. Improved insulin sensitivity, as seen with weight loss and exercise, can lead to increased adiponectin levels [50].

### 3.8. Adiponectin Receptors

Adiponectin shows its effects after binding to specific receptors, AdipoR1 and AdipoR2. Various tissues, including skeletal muscle, liver, and the cardiovascular system show expression of these receptors, which can affect the secretion of adiponectin [51]. Adiponectin receptors are expressed in skeletal muscle. When adiponectin binds to these receptors, it increases insulin sensitivity, which leads to improved glucose uptake by muscle cells and ultimately regulates blood glucose levels [52]. Adiponectin receptors are also expressed in the liver, which suppresses the production of excess glucose. Moreover, adiponectin promotes lipid breakdown in the liver, which prevents excessive fat accumulation and improves insulin sensitivity [53]. Adiponectin exerts anti-inflammatory actions on the cardiovascular system, which results in reducing inflammation in blood vessel walls and preventing atherosclerosis [54].

### 3.9. Aging

As individuals age, there is a decrease in the activity of brown adipose tissue, a decline in sex hormone levels, and an expansion of abdominal adipose tissue. This is accompanied by a shift of lipids from the subcutaneous fat compartment to the visceral fat compartment. This eventually results in reduced production of adiponectin [55].

### 3.10. Therapeutic Interventions

Certain medications and lifestyle interventions can affect adiponectin levels. Anti-diabetic drugs such as thiazolidinediones (TZDs) and metformin can increase adiponectin levels [56,57]. Thiazolidinediones (TZDs) elevate adiponectin levels, improving insulin sensitivity, enhancing AMPK activation, and reducing gluconeogenesis in the liver [24]. A meta-analysis showed that the administration of metformin led to a notable rise in serum adiponectin levels [58].

## 4. Adiponectin and Diabetes

**Figure 3:**
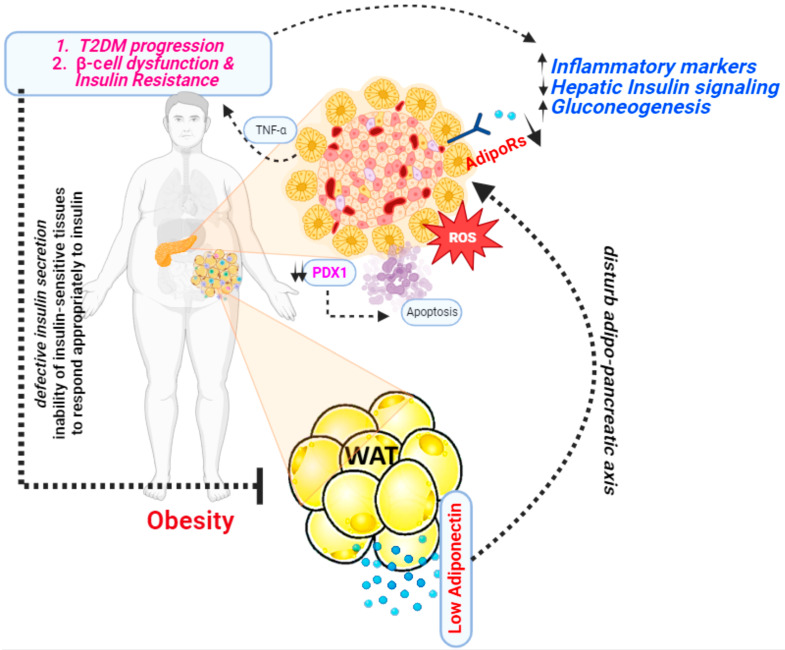
A presentation illustrating the mechanism through which adiponectin functions as an antidiabetic agent.

In 1995, a group of researchers from the Whitehead Institute for Biomedical Research, Cambridge, Massachusetts first discovered adiponectin [12]. This was a significant discovery because at the time, adipose tissue was primarily viewed as a passive energy storage site. Studies revealed that adiponectin plays an important role in modulating insulin sensitivity. Higher levels of adiponectin have been linked to better insulin sensitivity, whereas lower levels have been linked to insulin resistance [59]. Several studies have shown a significant correlation between adiponectin levels and diabetes (Figure 3).

## 5. Insulin Resistance and Adiponectin

Insulin resistance has a hereditary component that is not fully understood and is frequently passed down through generations. Furthermore, obesity has a significant hereditary component that inevitably worsens insulin resistance. As a result, obesity and insulin resistance are often present for many years before additional alterations such as high blood pressure, dyslipidemia, T2DM, and cardiovascular disease develop [19,60]. It was discovered that, in both mice and humans, a reduction in adipose tissue leads to increased levels of circulating triglycerides and fatty acids. The presence of insulin resistance provides further substantiation for the pivotal role played by adipose tissue in governing systemic metabolism by lipid storage [61,62,63]. Additionally, the appropriate release of adipokines like leptin and adiponectin, which improve insulin sensitivity, depends on the amount of adipose tissue. Lipodystrophies affect adipokine secretion in humans and mice.

The first study to demonstrate that adiponectin actively affects insulin sensitivity was reported in 2001. A C-terminal globular adiponectin fragment can lower plasma glucose levels by boosting fatty acid oxidation in muscle [14,64,65]. Globular adiponectin appears to function in conjunction with AMP-activated protein kinase (AMPK) (and later by inhibiting acetyl-CoA carboxylase) and PPAR-α (peroxisome proliferator-activated receptor alpha) to create its metabolic action in the muscles. Ceramidase silencing can inhibit AMPK phosphorylation in C2C12 myotubes, indicating a function for sphingolipid metabolism with adiponectin signaling in this tissue. Adiponectin binding increases glucose uptake (through GLUT4 translocation) and non-oxidative glycolysis while decreasing intramyocellular triacylglycerol concentration and boosting fatty acid oxidation. Furthermore, adiponectin affects the number of mitochondria and the kind of oxidative fibers [64]. Adiponectin’s actions on skeletal muscles are diminished in disease situations. Obese and insulin-resistant rats had poorer binding of globular and full-length adiponectin, which may be attributed to a lower density of adiponectin receptors. Human investigations, on the other hand, have not shown changed levels of Adipor1/Adipor2 mRNA related to insulin resistance states [66].

## 6. Apoptosis and Adiponectin

Liu et al. reported that, by stimulating the AdipoR1/AMP-activated protein kinase (AMPK) signal pathway, adiponectin decreased early apoptotic cells and prevented the mitochondrial apoptosis process in adipocyte culture. Furthermore, PPAR linked to the ATF2 promoter area and suppressed ATF2 transcription. ATF2 transcriptional suppression was associated with adiponectin’s ability to prevent apoptosis in adipocytes [67]. Zuo et al. reported that adiponectin suppresses inflammation and reduces apoptosis caused by excessive hyperglycemia by inhibiting the TLR4/NF-B signaling pathway [68]. The initiation of apoptosis by adiponectin is mainly facilitated through AdipoRs, which initiate the activation of caspase family proteins (caspase-3, -8, -9) [69,70].

Treatment of beta-cell line INS-1 cells with a cytokine combination (IL-1b/IFN-c) or palmitic acid strongly promoted apoptosis, which could be greatly suppressed by gAPN via caspase-3 inhibition without altering NF-_k_B [71]. Lin et al. reported Adiponectin cotreatment partially reversed high glucose-induced INS-1 cell death, malfunction, and decrease in insulin gene expression, which was mediated at least in part by transiently activating the AMPK signaling system [69]. Adiponectin has also been identified to modulate several additional molecular pathways involved in apoptosis. This contains the Bcl-2 family of proteins, which are critical in managing the balance of pro-apoptotic (cell death-promoting) and anti-apoptotic (cell death-inhibiting) signals inside the cell. Adiponectin has the ability to modulate the expression and activity of Bcl-2, Bax, and Bak proteins [72].

Adiponectin may also have an effect on the tumor suppressor protein p53, which is important in starting apoptosis in response to cellular stress or injury. The action of adiponectin on p53 may contribute to its proapoptotic effects [73,74]. Adiponectin has also been demonstrated to affect the iNOS/ROS/RNS pathways. All molecules involved in cellular signaling and stress responses are iNOS (inducible nitric oxide synthase), ROS (reactive oxygen species), and RNS (reactive nitrogen species). The regulation of these pathways by adiponectin may contribute to its proapoptotic effects [75].

## 7. β-Cell Function and Adiponectin

**Figure 4:**
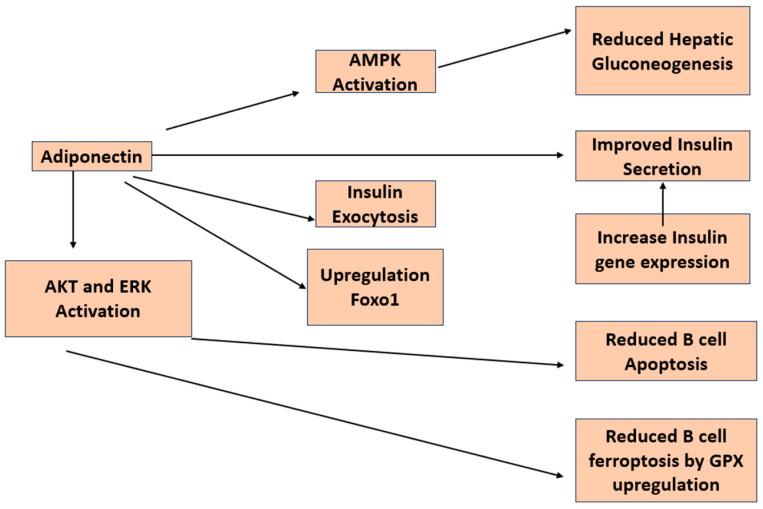
Diagram depicting the investigated pathways illustrating the impacts of adiponectin on pancreatic β-cells.

There have been a number of studies looking at the direct impact of adiponectin on insulin secretion in β-cells (Figure 4). A group of researchers from the University of Tokyo reported that adiponectin enhances insulin release from isolated mouse islets by promoting the exocytosis of insulin granules, with no discernible impact on ATP production, KATP channels, membrane potential, calcium influx, or activation of AMPK [76]. An additional investigation demonstrated that adiponectin safeguards β-cells from apoptosis induced by prolonged serum deprivation and glucotoxicity. These outcomes are facilitated by the activation of both MEK-extracellular signal-regulated kinase (ERK) 1/2 and PI3K-Akt pathways [77]. James E P et al. reported that globular adiponectin induces a notable enhancement in cell viability, dependent on ERK1/2 signaling, along with a substantial rise in Pdx-1 expression in rat β-cell lines [78]. Adenosine monophosphate-activated protein kinase (AMPK) is triggered by adiponectin, leading to the direct phosphorylation and subsequent inhibition of acetyl-CoA carboxylase activity in β-cells [79].

Adiponectin knockout mice exhibit compromised glucose tolerance, even in the presence of normal or lower-than-normal insulin levels [80]. Transgenic ob/ob mice expressing the globular domain of adiponectin demonstrate heightened insulin sensitivity and elevated insulin secretion in comparison to nontransgenic mice [32,81,82] In vivo experiments conducted in C57BL/6 mice revealed that intravenous administration of adiponectin leads to an augmentation in insulin secretion [76].

An observational study involving Asian children found that adiponectin levels exhibit an inverse relationship with body weight, body mass index, and proinsulin levels in both boys and girls. Moreover, in girls, there is an inverse association between adiponectin levels and insulin concentration as well as the homeostasis model assessment of insulin resistance (HOMA-IR) [83]. Studies have demonstrated a positive correlation between adiponectin levels and insulin sensitivity. Conversely, there is an inverse correlation between adiponectin levels and fasting proinsulin concentration, as well as the proinsulin-to-insulin ratio, which serves as a marker of β-cell failure [84]. Furthermore, it has been suggested that the decrease in adiponectin levels is longitudinally linked with a reduced ability of β-cells to compensate for insulin resistance in women with a history of gestational diabetes [85]. In overweight Hispanic adolescents, a cross-sectional study affirmed that both leptin and adiponectin are individually linked to insulin sensitivity, while they do not exhibit an association with insulin secretion [86].

## 8. Oxidative Stress and Adiponectin

The production of reactive oxygen species (ROS) leads to oxidative stress, causing a range of cellular and molecular alterations, including dysfunction in mitochondria, which disrupt normal physiological processes in the body [87,88,89,90]. While oxidative pathways are crucial in mitochondrial-mediated processes, the exact molecular mechanisms responsible are still unclear. The compromised mitochondrial function is evident in insulin resistance across different cell types. Furthermore, ongoing research is unraveling the roles of the master antioxidant pathway involving nuclear factor erythroid 2-related factor 2 (Nrf2), Kelch-like ECH-associated protein 1 (Keap1), and antioxidant response elements (ARE) in elucidating various molecular pathways associated with diabetes. There are two contrasting theories regarding adiponectin and oxidative stress. While some studies suggest that adiponectin lowers oxidative stress levels, others indicate that elevated oxidative stress can diminish adiponectin production.

Kadowaki and colleagues’ findings revealed that oxidative stress was elevated in mice lacking AdipoR1 and AdipoR2, offering compelling evidence that the adiponectin-AdipoRs pathway plays a pivotal role in reducing oxidative stress [29]. In a mouse model of kidney disease, the absence of adiponectin resulted in heightened albuminuria and elevated expression of genes associated with oxidative stress [91]. In experiments conducted on cultured murine pre-adipocytes (3T3-L1), it has been observed that oxidative stress leads to a reduction in the secretion of adiponectin [92]. In 2006, Chen et al. conducted experiments using cultured pre-adipocytes (3T3-L1) and discovered that ROS decreased the expression of adiponectin mRNA. In a separate study, 3T3-L1 pre-adipocytes were subjected to oxidative stress by introducing H_2_O_2_ or glucose oxidase into the incubation medium [93]. In 2015, Pan et al. discovered that H_2_O_2_ decreased adiponectin production by 3T3-L1 adipocytes by a factor of 2, and led to a threefold increase in the synthesis of TNF-α and IL-6. The oxidative stress induced by the addition of H_2_O_2_ to the incubation medium of 3T3-L1 pre-adipocytes resulted in elevated mRNA levels of leptin, IL-6, and MCP-1 (monocyte chemoattractant protein 1), along with increased secretion of these proteins by adipocytes. Notably, there was an almost threefold increase in the secretion of IL-6 [94,95].

On the other hand, adiponectin suppresses the harmful effects of oxidative stress. In an AMPK-independent manner, adiponectin reduces the production of oxidative stress by inhibiting inducible nitric oxide synthase and suppressing the expression of gp91phox, a subunit of NADPH oxidase [96]. Another report shows that adiponectin can suppress oxidative stress in the endothelium of hyperlipidemic rats [97].

## 9. Anti-Inflammatory Functions of Adiponectin

Numerous metabolic strains that contribute to insulin resistance and T2DM also trigger the activation of inflammation- and stress-related enzymes, namely IκB kinase-β (IKKβ) and JUN N-terminal kinase (JNK). This implies that these kinases likely play pivotal roles in the development of these disorders [98]. Specifically, IKKβ initiates the activation of the transcription factor nuclear factor-κB (NF-κB), and obesity leads to heightened expression of NF-κB-regulated genes, such as pro-inflammatory cytokines, in both the liver and adipose tissue [98]. These cytokines, encompassing TNF, IL-6, and IL-1β, can potentially induce insulin resistance in the originating tissues like the liver and adipose tissue [99]. Furthermore, they may be disseminated through the circulation, exerting their effects on more remote locations such as vessel walls, skeletal and cardiac muscle, the kidneys, and circulating leukocytes [100]. The involvement of IL-6 signaling in insulin resistance has sparked controversy, displaying occasional paradoxical effects [101]. Elevated levels of circulating IL-6 and CRP, which is stimulated by IL-6 in the liver, are observed in obesity and serve as predictive markers for type 2 diabetes in predisposed individuals [102]. While hepatic and adipose tissue production of IL-6 is believed to contribute to insulin resistance, its generation in skeletal muscle, particularly during intense exercise, is considered advantageous [103]. The examination of mice with specific deletion of the IL-6 receptor in hepatocytes has further fueled the debate, as these mice appear to be shielded from both local and systemic insulin resistance [104].

A number of experimental studies with genetic loss-of-function manipulations indicate that ablation of adiponectin contributes to diet-induced insulin resistance, increased vascular remodeling in response to injury, and severe cardiac damage under ischemic conditions [105]. A sequence of in vitro experiments has shown that adiponectin has the capacity to impede the production and effects of TNFα, which is a pivotal proinflammatory cytokine. This effect has been observed in different types of cells, including cardiac and vascular cells [106]. Devaraj et al. provided evidence that adiponectin can inhibit the production of CRP induced by high glucose levels. This inhibition occurs through adiponectin’s capacity to suppress the activation of nuclear factor-κ B (NF-κB). These findings align with earlier research that demonstrated adiponectin’s ability to mitigate TNF-α-induced NF-κB activation in endothelial cells. This, in turn, leads to decreased expression of cell adhesion molecules and interleukin (IL)-8 [107].

## 10. Single-Cell Data from db/db Mice Pancreatic Islet Cells

Evaluating the expression of AdipoR1/R2 in single-cell pancreatic islet RNAseq datasets related to Early and Late Diabetes mellitus (DM) offers a promising avenue for researchers to gain deeper insights into the potential role of APN/AdipoRs signaling in DM. This investigation encompasses its influence on genes associated with various critical aspects, including apoptosis, β-cell function, oxidative stress, inflammation, and cellular growth within pancreatic islet cells. This newfound knowledge may significantly contribute to the development of more effective treatments for type 2 diabetes mellitus. To date, none of the literature on single-cell RNAseq presented the expression of APN/AdipoRs in the pancreatic islet cell population, underscoring the urgent need to elucidate the APN pathway genes throughout the Early and Late DM pathogenesis.

**Figure 5:**
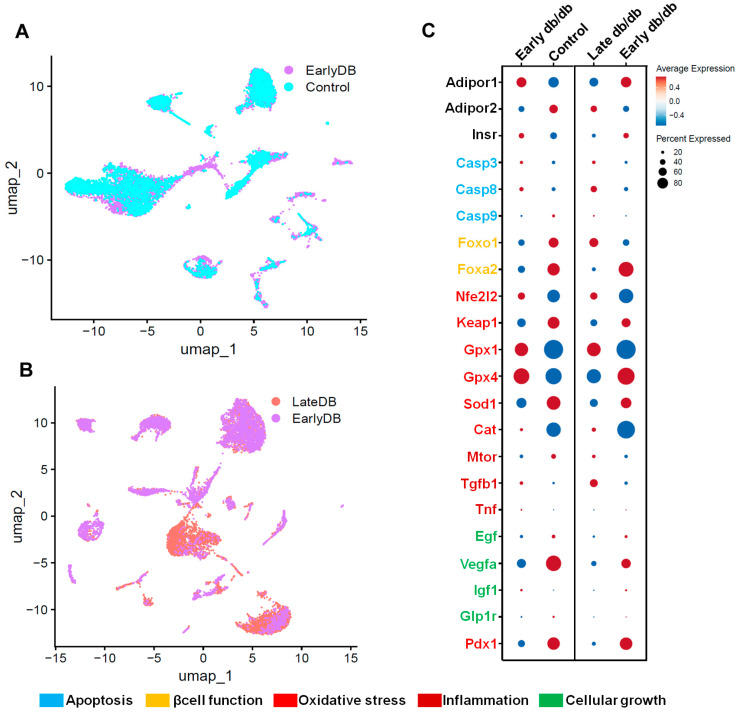
Single-cell UMAP visualization depicting the cellular landscape comparison of Early db/db vs. Control (**A**) and Late db/db vs. Early db/db (**B**) of pancreatic islet cells. The differential expression of critical genes in adiponectin signaling, apoptosis, β-cell function, oxidative stress, inflammation, and cellular growth are depicted in color indications (**C**).

To address our hypothesis, we acquired the single-cell data of pancreatic β islet cells from GEO (https://www.ncbi.nlm.nih.gov/geo/, accessed on 25 October 2023) using the accession number GSE165267, which is processed by Wei et al. [108]. This dataset was analyzed using Seurat software v4.1.1 [109] implemented in R v4.2.1. We conducted an in-depth analysis of the pancreatic β islet cellular landscape by plotting UMAP (Uniform Manifold Approximation and Projection) in the comparison of Early db/db vs. Control (Figure 5A) and Late db/db (Figure 5B). From this analysis, a significant reduction in cell population was found in Late db/db when compared to Early db/db. Whereas, no obvious changes in cell population were found in Early db/db when compared to the Control. Furthermore, the differential gene expression was computed between these groups and represented as a dot plot as depicted in Figure 5C.

In our observations, we noted a decrease in adiponectin receptor levels compared to the control group, coinciding with the progression of diabetes in the pancreatic islet cells of diabetic mice. This reduction in adiponectin receptor levels was associated with an upregulation of apoptosis-related genes, specifically Casp3 and Casp9, and a downregulation of antioxidant genes such as Gpx4, Gpx1, and Sod1. Additionally, there was an upregulation of inflammatory genes including Mtor, Tgfb1, and Tnf. These findings align with our earlier hypotheses and support the proposed pathway involving the role of adiponectin in T2DM.

## 11. Future Directions and Challenges

A multimodal strategy is required for future developments in adiponectin-based diabetic therapy. Adiponectin receptor agonists [110,111] are being developed by researchers in order to imitate the positive effects of adiponectin on insulin sensitivity and glucose metabolism [24]. Gene treatments have the potential to increase adiponectin expression or activity in diabetics. The goal of pharmaceutical treatments is to find molecules that can increase adiponectin production from adipose tissue. Understanding how food and exercise affect adiponectin levels is also an important area of research. Combinatorial techniques, personalized medicine approaches, biomarker research, and rigorous clinical trials are all critical components in enhancing the promise of adiponectin-based therapeutics for improving metabolic health in diabetic patients. It is critical to understand that any new therapies will need to go through extensive testing and regulatory processes before they can be used in clinical trials. However, adiponectin’s potential as an antidiabetic drug is still undergoing clinical trials, and the outcomes of these trials thus far have been highly encouraging.

## 12. Conclusions

In conclusion, the significance of adiponectin in diabetes management cannot be overstated. Its pivotal role in regulating glucose metabolism and insulin sensitivity highlights its potential as a promising therapeutic target. As research in this sector advances, tapping the full potential of adiponectin may lead to novel and successful diabetes therapies. Further research, including clinical trials and in-depth molecular investigations, will be critical in achieving the full therapeutic potential of this unique hormone. With ongoing effort and scientific study, the road to improving diabetes management with adiponectin-based therapy holds enormous potential.

## Data Availability

No new data were created.
